# Learning Cell-Type-Specific Gene Regulation Mechanisms by Multi-Attention Based Deep Learning With Regulatory Latent Space

**DOI:** 10.3389/fgene.2020.00869

**Published:** 2020-09-30

**Authors:** Minji Kang, Sangseon Lee, Dohoon Lee, Sun Kim

**Affiliations:** ^1^Bioinformatics Institute, Seoul National University, Seoul, South Korea; ^2^Interdisciplinary Program in Bioinformatics, Seoul National University, Seoul, South Korea; ^3^Department of Computer Science and Engineering, Institute of Engineering Research, Seoul National University, Seoul, South Korea

**Keywords:** gene regulation mechanism, gene regulatory network, multi-omics, deep learning, cell-type-specific

## Abstract

Epigenetic gene regulation is a major control mechanism of gene expression. Most existing methods for modeling control mechanisms of gene expression use only a single epigenetic marker and very few methods are successful in modeling complex mechanisms of gene regulations using multiple epigenetic markers on transcriptional regulation. In this paper, we propose a multi-attention based deep learning model that integrates multiple markers to characterize complex gene regulation mechanisms. In experiments with 18 cell line multi-omics data, our proposed model predicted the gene expression level more accurately than the state-of-the-art model. Moreover, the model successfully revealed cell-type-specific gene expression control mechanisms. Finally, the model was used to identify genes enriched for specific cell types in terms of their functions and epigenetic regulation.

## 1. Introduction

Epigenetic gene regulation is a major control mechanism of gene expression. Histone modifications one of the most versatile modes of chromatin regulation among diverse epigenetic regulatory mechanisms are defined as covalent modifications of a set of specific amino acids at N-terminal tails of histone proteins. Combinations of the type of amino acids and their modifications constitute “histone codes” that are distributed across the genome and are known to regulate overall chromatin states. On the other hand, DNA methylation occurs directly at the cytosine bases of DNA and regulates gene expression in part by altering the binding affinity of most of the transcription factors. Besides the individual effect of each epigenetic modification, the complexity of epigenetic gene regulation mostly arises from the crosstalk between the different types of epigenetic modifications. For example, positive interplay between histone marks (1) H2BK120u1 and H3K4me3, and (2) H3K4me3 and H3/H4 acetylation (Zhang et al., 2015) is an example of the complex epigenetic regulation. Furthermore, some histone modifications are known to be associated with DNA methylation (Cedar and Bergman, 2009). *De novo* DNA methyltransferases, DNMT3A and DNMT3B, are known to physically interact with specific histone marks, H3K36me3 and H3K4me0, through their internal PWWP and ADD domain, respectively. Methyl-CpG-binding domain (MBD) proteins have been reported to “read” methylated CpG, and recruit chromatin-modifying complexes such as SWI/SNF components (Fatemi and Wade, 2006). Subtle epigenetic interactions between different types of histone modifications and DNA methylation can therefore be regarded as a major determinant of the general chromatin structure of cells that govern the accessibility of transcription factors to the chromatin.

Given the essential role of epigenetic alterations in regulating gene expression, a number of studies on modeling the regulatory effects of these epigenetic markers have been performed. However, existing modeling methods utilize only a single epigenetic marker. Some studies have investigated the role of histone marks in the context of gene regulation. DeepChrome (Singh et al., 2016) used a Convolutional Neural Network based model to model gene regulation. It was the first deep learning approach to predict the gene expression level, and it captured local characteristics of histone marks. Another study, AttentiveChrome (Singh et al., 2017), proposed a hierarchy of multiple Long Short-Term Memory modules with an attention mechanism to predict gene expression levels. AttentiveChrome predicted gene expression more accurately than DeepChrome, and it showed which histone marks or which gene loci were used, using an attention mechanism. Both studies used individual deep learning approaches to understand gene regulation but utilized histone marks only. There have also been studies to identify relationships between genome-wide DNA methylation and gene expression. Wagner et al. (2014) investigated the relationships between DNA methylation and the gene expression profile of primary fibroblast samples from 62 individuals. More recently, Zhong et al. (2019) predicted gene expression using DNA methylation in human populations, using linear regression-based methods. Recent studies investigated the relationships between mutation and gene expression. Zeng et al. (2017) used a linear regression-based model to predict gene expression with cis-SNPs. Xie et al. (2017) examined the effectiveness of a deep auto-encoder to predict the gene expression profile measured in yeast with SNP. These prior studies on gene expression prediction revealed relationships between gene expression and a single epigenetic marker of histone marks, DNA methylation, or SNP. However, these studies were not designed to model complex transcriptional control mechanisms involving the interplay of various epigenetic regulatory modules.

We therefore introduce an *explainable* deep learning model with a multi-attention network for epigenetic regulation mechanisms. Our model integrates multiple markers such as histone marks, DNA methylation, and transcription factors and explains the complex interactions between the molecular regulators. The attention network modules of our model allow human experts to understand the gene regulation mechanisms. Moreover, the model characterizes cell-type-specific gene regulation mechanisms for 18 cell lines, based on the weights of the Multi-Attention network. In summary, the proposed model provides a better understanding of cell-type-specific gene regulation.

## 2. Materials and Methods

**Figure 1:**
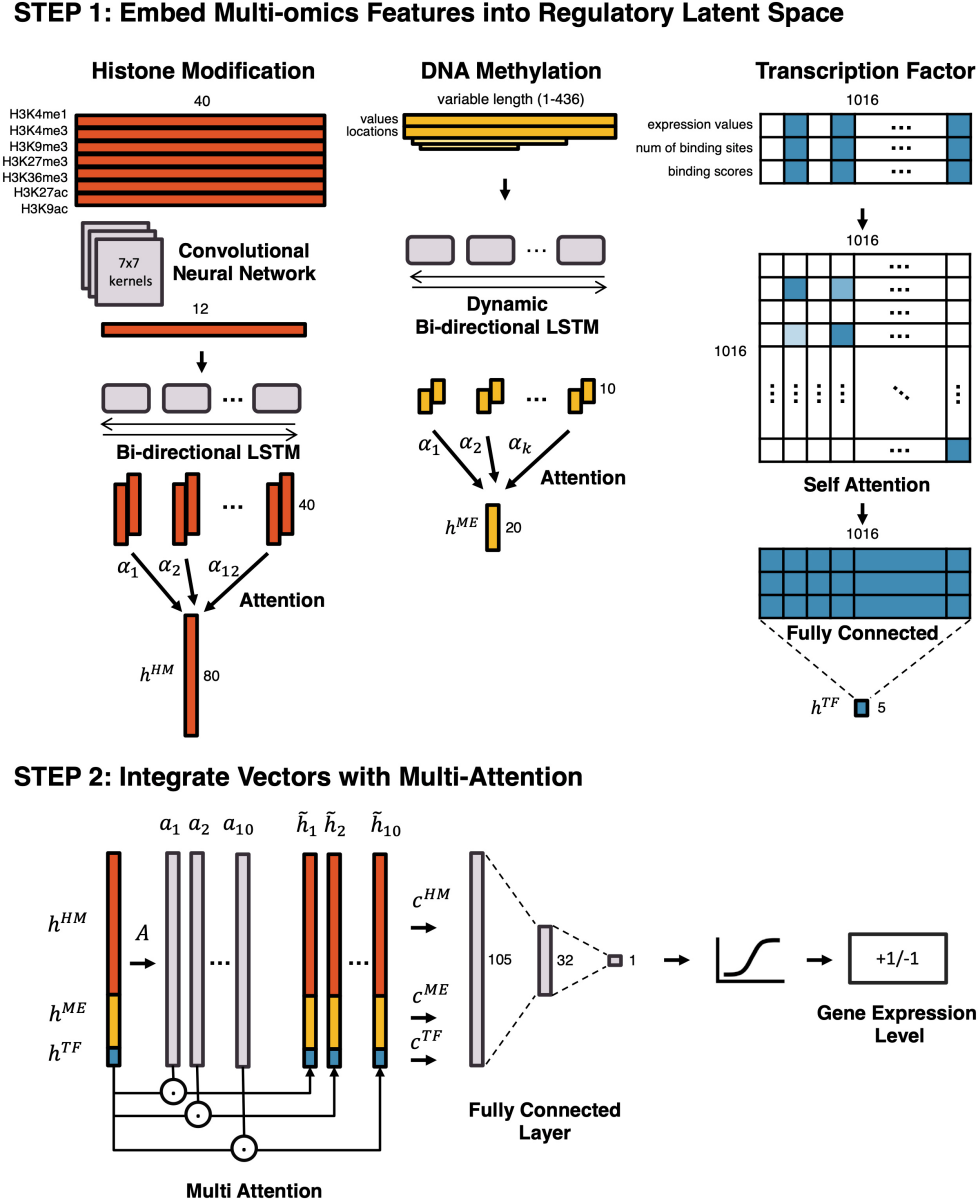
An overview of the proposed model. To predict gene expression level and to model the regulation mechanism, a Multi-Attention based deep learning model with regulatory latent space is designed. It consists of two steps: (1) embedding multi-omics features into a regulatory latent space, and (2) integrating latent vectors with a Multi-Attention network. In the first step, different deep learning architectures are utilized to reflect the characteristics of each omics feature. By omics-specific layers, multi-omics features are transformed into latent vectors in the regulatory latent space. In the second step, the latent vectors are integrated by a Multi-Attention network. The attention weights of multi-omics features represent their effects on the gene regulation.

We propose a two-step ensemble deep learning model for gene expression prediction and the architecture is illustrated in Figure 1. At the first layer of the model, separate neural networks vectorize epigenetic and transcriptional markers with different strategies, and then at the second layer, output vectors from the first layer are integrated by a Multi-Attention network. To predict gene expression, we used the same outputs previously used in DeepChrome (Singh et al., 2016) and AttentiveChrome (Singh et al., 2017). All genes are divided into highly expressed genes (HEG) and lowly expressed genes (LEG) according to their expression levels, which formulates the problem as a binary classification task.

To begin, separate models embed histone marks, DNA methylation, and transcription factors into a regulatory latent space. First, histone marks are embedded into the latent space by a Convolutional Neural Network (CNN) followed by a Bi-directional Long Short-Term Memory (LSTM) network with attention. Second, DNA methylation is vectorized by a Dynamic Bi-directional LSTM with attention. Lastly, a Self-Attention Network (SAN) embeds the transcription factors. After embedding features in three vectors, a Multi-Attention network combines these vectors to predict whether a gene would be highly expressed or lowly expressed. While the end-to-end model predicts the gene expression level as a whole, the Multi-Attention network determines which types of epigenetic markers are most influential for controlling gene expression and how epigenetic features interact with each other in each cell type.

We used datasets from the Roadmap Epigenomics Projects (Kundaje et al., 2015) to predict the gene expression level of 18 cell lines, for which data measuring levels of histone marks, DNA methylation, and transcription factors are available (Table 1, Supplementary Figure 1). The epigenetic and transcriptional markers near the transcription start site (TSS) mainly involve in gene expression. We therefore focused on the gene region of 4,000 base-pair (bp) around the TSS for histone markers or DNA methylation and 200 bp around the TSS for transcription factors. To implement the model, we used Pytorch, an open-source machine learning library based on Python. Implementation of our model can be found at Github (https://github.com/pptnz/deeply-learning-regulatory-latent-space).

**Table 1:** Cell line data used in this study.

**Cell line**	**Group**	**Standardized epigenome name**
E003	ESC	H1 Cells
E004	ES-deriv	H1 BMP4 derived mesendoderm cultured cells
E005	ES-deriv	H1 BMP4 derived trophoblast cultured cells
E006	ES-deriv	H1 derived mesenchymal stem cells
E007	ES-deriv	H1 derived neuronal progenitor cultured cells
E011	ES-deriv	hESC derived CD184+ endoderm cultured cells
E016	ESC	HUES64 cells
E038	Blood & T-cell	Primary T helper naive cells from peripheral blood
E047	Blood & T-cell	Primary T CD8+ naive cells from peripheral blood
E066	Tissue & Primary cell	Liver
E087	Tissue & Primary cell	Pancreatic islets
E114	Cancer cell line	A549 EtOH 0.02pct lung carcinoma cell line
E116	Cancer cell line	GM12878 lymphoblastoid cells
E117	Cancer cell line	HeLa-S3 cervical carcinoma cell line
E118	Cancer cell line	HepG2 hepatocellular carcinoma cell line
E119	Tissue & Primary cell	HMEC mammary epithelial primary cells
E120	Tissue & Primary cell	HSMM skeletal muscle myoblasts cells
E123	Cancer cell line	K562 leukemia cells

In the following sections, deep learning models for each of the epigenetic and transcriptional markers are explained.

### 2.1. Embedding Histone Marks

We used seven core histone marks: H3K4me1, H3K4me3, H3K9me3, H3K27me3, H3K36me3, H3K27ac, and H3K9ac. Among 31 histone marks in the Roadmap Epigenomics Projects, the seven core histone marks had been profiled and investigated the most. Each of the seven histone marks were profiled for more than 62 cell lines, whereas other histone marks were profiled for less than 24 cell lines (Supplementary Figure 2). To investigate cell-type-specific gene regulation mechanisms, we used the seven histone marks with abundant cell line data.

To vectorize the histone marks, we used CNN, followed by Bi-directional LSTM with an attention mechanism. CNN is a deep learning architecture proposed for extracting local features of various sizes in two-dimensional images (Min et al., 2016). In this model, CNN captures local patterns of the seven histone marks. RNN is a deep learning architecture with a cyclic structure, which has caught the limelight in natural language processing fields (Min et al., 2016). LSTM is one of the architectures of the Recurrent Neural Network, proposed for considering long-term dependencies (Gers et al., 2000). Unlike other RNN architectures, LSTM has a forget gate, which allows the model to forget irrelevant parts of a sequence and deal with a long sequence. In our model, LSTM captures sequential patterns. The attention mechanism reveals important gene loci.

The histone marks in a gene region of 4,000 bp around TSS are divided into 40 bins with a bin size of 100 bp. On each bin, log read counts are calculated for each histone mark, respectively. The preprocessed histone mark matrix of size 7 × 40 is fed into a CNN that consists of a convolutional layer, a batch normalization layer, and a 1D max-pooling layer. In the convolutional layer, 100 kernels of size 7 × 7 are used, so that a vector of size 1 × 34 is produced. In the max-pooling layer, a kernel with size 3 and stride 3 is used with left and right padding. Afterward, the output vector of the CNN is fed into the Bi-directional Long Short-Term Memory (LSTM) with attention, producing a *h*^*HM*^ of size 80.

### 2.2. Embedding DNA Methylation

We used methylation values at all CpG sites within up/down-stream of 2,000 bp from TSS. DNA methylation is vectorized by a Dynamic Bi-directional LSTM with attention. The number of CpG sites vary for different genes. Thus, the “Dynamic” LSTM deals with the variable number of CpGs, and the “Bi-directional” LSTM considers both directions of the DNA strands. Dynamic Bi-directional LSTM produces the output vector *h*^*ME*^ of a fixed size 20.

### 2.3. Embedding Transcription Factors

We first selected candidate binding transcription factors (TFs) for each gene, based on prior knowledge of human transcription factors in Lambert et al. (2018), and the motif detection tool, HOMER (Heinz et al., 2010). We utilized TFs that have their binding sites within the region of 200 bp around the TSS. Based on this configuration, an input matrix for TFs is processed as a matrix of size 3 x 1016. Three rows of an input matrix represent TF expression values, the number of binding sites, and the binding scores of TFs by HOMER. One-thousand-and-sixteen columns of the matrix represent human transcription factors. Except for the candidate binding transcription factors, all columns are masked to zero.

Since the data of transcription factors are discrete rather than sequential, CNN or LSTM cannot be employed. Thus, a Self-Attention Network (SAN) is used to embed the input matrix in vector a *h*^*TF*^ of size 5. As a result of SAN, the attention weight matrix is produced, providing vital information about relationships and interactions between transcription factors.

### 2.4. Integrating Latent Vectors

To integrate latent vectors, we used the Multi-Attention Block from the Multi-Attention Recurrent Network (MARN) (Zadeh et al., 2018). MARN was proposed for the comprehension of human communication with multi-modal data (language modality, vision modality, and acoustic modality). As it was designed to deal with data with different characteristics, MARN is suitable for dealing with three latent vectors from different multi-omics data.

First, all three latent vectors *h*^*HM*^, *h*^*ME*^, and *h*^*TF*^ are concatenated. The concatenated vector *h* is fed into a fully connected layer *A*. Multiple attention weights *a*_1_, *a*_2_, ..., *a*_*k*_ are then produced, where *k* is the number of attentions. The k attention weights are multiplied to the concatenated vector, the vectors ${\tilde{h}}_{1},{\tilde{h}}_{2},...,{\tilde{h}}_{k}$ are produced by element-wise multiplication of the concatenation and the *k* attention weights as ${\tilde{h}}_{i}=h⊗a_{i}$.

Finally, a fully connected layer produces the predicted labels, which represents whether a gene is highly expressed (HEG, +1) or lowly expressed (LEG, -1).

## 3. Results

To evaluate our proposed model, we split 18,070 genes into four-folds for each cell line. The first and second folds were used as a test and validation set, respectively, and the remaining two folds were used as a training set. Every result is averaged from a 4-fold cross-validation.

### 3.1. Performance Evaluation of Models With Histone Modification Only

We set a baseline with the state-of-the-art method for gene expression level prediction, AttentiveChrome (Singh et al., 2017). As AttentiveChrome was designed for histone marks only, instead of multiple epigenetic features, we trained both our model and AttentiveChrome using only seven histone marks for a fair comparison. We evaluated them with two metrics. (1) First, we performed a classification task on whether a gene is highly or lowly expressed in that cell line. (2) Second, gene expression value prediction was performed in terms of rank concordance between the gene expression values and the final output values of the model.

**Figure 2:**
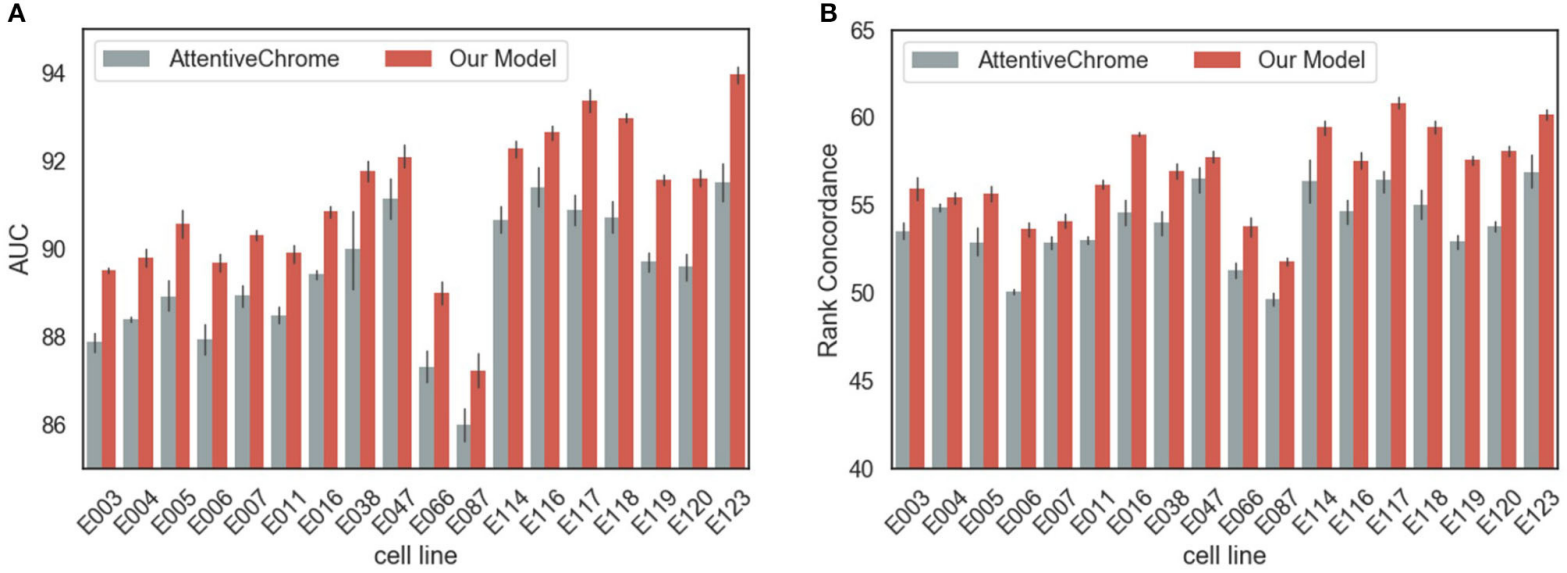
Performance evaluation of models in predicting the gene expression levels. The performance of our model surpassed that of a baseline model, AttentiveChrome, in both criteria of **(A)** AUC and **(B)** Rank Concordance for two models for every cell line.

In terms of AUC, rank concordance, and AUPR, our model outperformed AttentiveChrome for every cell line (Figure 2, Supplementary Figure 3). On average, the proposed model achieved 91.05% of AUC, while AttentiveChrome achieved 89.72%. Moreover, the model demonstrated its robustness by showing higher rank concordances between the gene expression value and the final output of the model. Our model showed 56.83% of rank concordance on average, while AttentiveChrome showed only 53.85%. We conjecture that the performance difference is due to the difference in model architectures. AttentiveChrome first uses an individual LSTM structure for each histone mark, and then integrates histone marks with an additional LSTM. On account of the individual LSTM, the interactions of numerous types of histone marks are likely to be neglected. Consequently, AttentiveChrome was not successful in capturing the local characteristics of seven histone marks. On the other hand, our model used both CNN and LSTM to capture local and sequential features of histone marks in a single model. Our model is therefore suitable for modeling not only the roles of histone marks but also interactions among them.

### 3.2. Performance Evaluation of Models With Multi-Omics Markers

**Figure 3:**
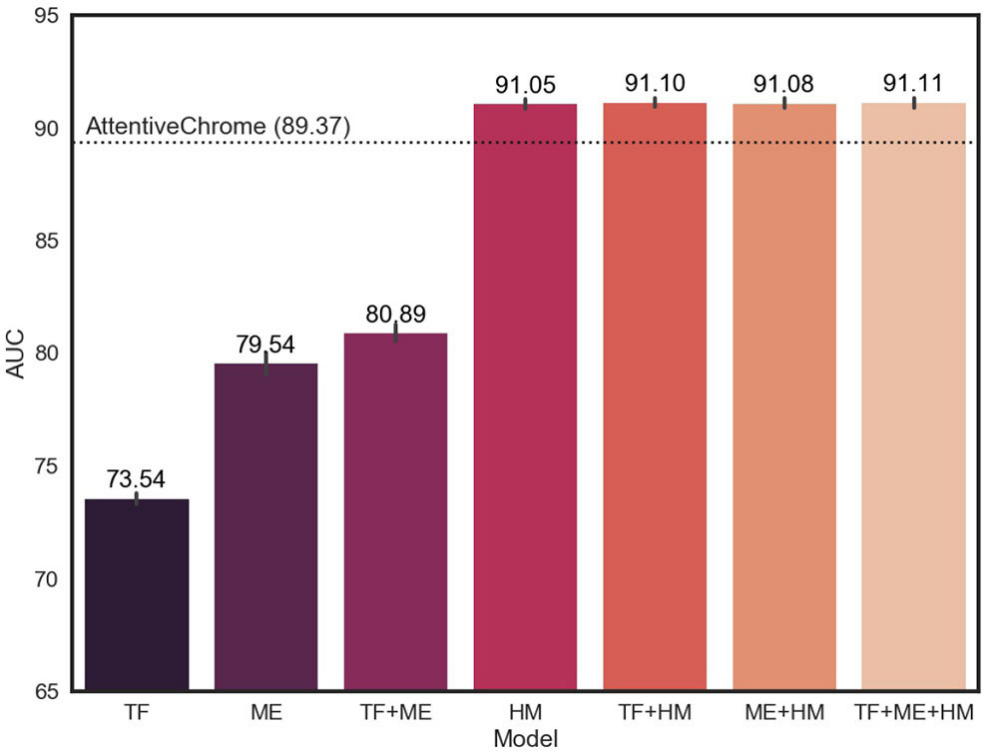
Average AUC of 18 cell lines for different subsets of multi-omics features. TF, ME, and HM stand for transcription factors, DNA methylation, and histone marks, respectively. The model showed improvement in AUC, adding multiple epigenetic markers.

Since our model is designed to utilize multi-omics biomarkers, we measured performance in terms of the average AUC and AUPR of our models that were trained on all possible combinations of multi-omics features (Figure 3, Supplementary Figure 4). The average AUC of the model improved when adding and integrating multi-omics features. In particular, the model with histone marks (HM, TF+HM, ME+HM, and TF+ME+HM) showed remarkable levels of AUC, exceeding the AUC of AttentiveChrome. This result is attributed to the fact that genes can be expressed if chromatins are opened, and thus histone marks are a major determinant of chromatin regulation.

### 3.3. Modeling Gene Regulation Mechanisms Using Multi-Omics Markers

**Figure 4:**
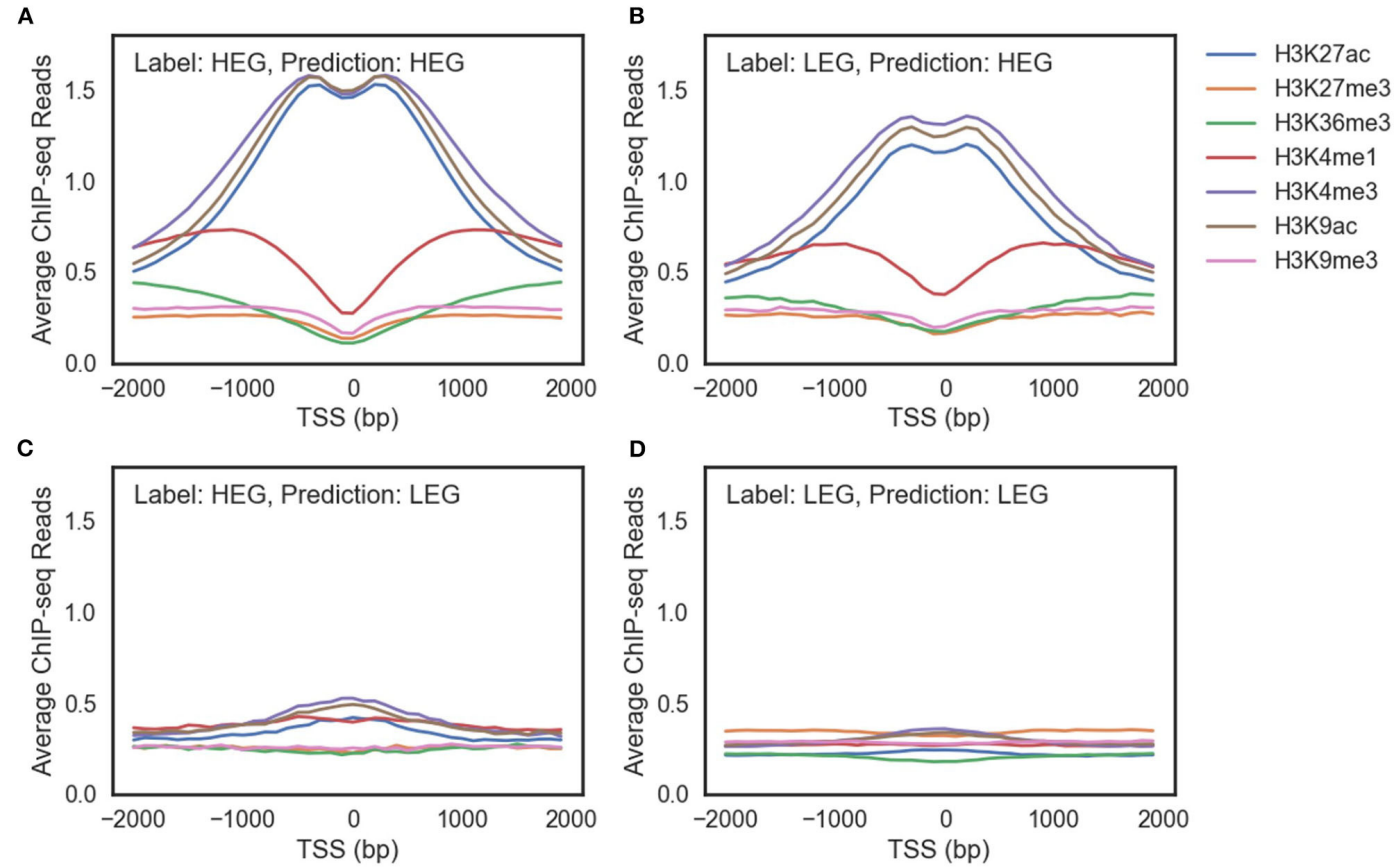
The average ChIP-seq reads of seven histone modification marks of **(A)** highly expressed genes (HEG) predicted as HEG. **(B)** lowly expressed genes (LEG) predicted as HEG. **(C)** HEG predicted as LEG. **(D)** LEG predicted as LEG.

Multi-omics markers are required to model gene regulation mechanisms. We focused on HeLa cell since its accuracy has been improved significantly by adding multiple markers (Supplementary Figure 5). In the HeLa cell, genes exist that cannot be predicted correctly using histone modification marks alone. Figure 4 shows the average ChIP-seq reads of histone modification marks of genes with the same labels and predictions of the HM model. The model prediction is consistent: genes with the same prediction curves have common characteristics regardless of their true labels.

There were three differences between genes predicted as highly expressed genes (HEG) (Figures 4A,B) and genes predicted as lowly expressed genes (LEG) (Figures 4C,D). First, ChIP-seq reads of the genes predicted as HEG had a larger scale than the genes predicted as LEG. Second, the genes predicted as HEG had a noticeable peak around TSS for each histone mark associated with the activation of genes (H3K27ac, H3K4me3, and H3K9ac). Third, the genes predicted as HEG had a higher value of H3K4me1 around TSS, which is related to enhancers.

There are LEG even with an open chromatin state (Figure 4B) and HEG with weak signals of activation histone marks (Figure 4C). A model that only uses histone marks is limited in both predicting the gene expression level and characterizing the gene regulation mechanisms. In other words, multiple epigenetic markers such as DNA methylation and transcription factors are required to understand the complex gene regulation mechanisms.

**Figure 5:**
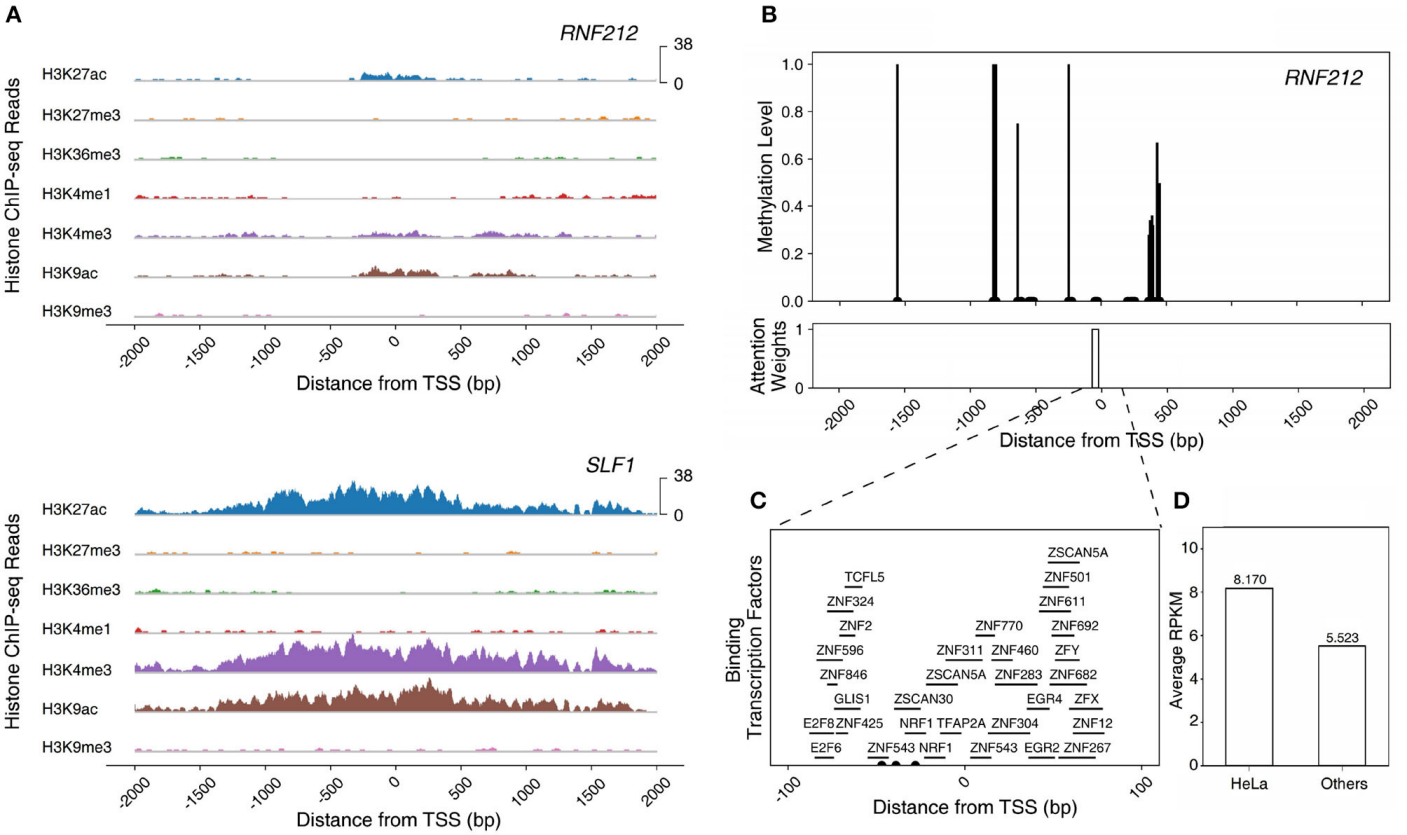
Modeling the epigenetic regulation mechanism of *RNF212* in HeLa cell line. **(A)** The ChIP-seq reads of histone modification marks of *RNF212* and *SLF1*. **(B)** The DNA methylation levels and attention weights of *RNF212*. **(C)** The candidate binding transcription factors of the promoter region of *RNF212*. **(D)** The average expression value of the binding transcription factors of *RNF212* in HeLa cell line and other cell lines.

*RNF212*, one of the enriched genes in the HeLa cell, epitomizes a gene that can be fully understood only by the multi-omics model, especially the TF+ME+HM model. The gene is a highly expressed gene but the model with histone marks alone failed to predict the gene expression level. This is due to the weak activation of histone marks. The intensities of histone marks associated with the activation of genes (H3K27ac, H3K4me3, and H3K9ac) were much smaller than those of other HEGs (i.e., *SLF1*) (Figure 5A). However, the gene was predicted correctly by the model with three epigenetic markers (TF+ME+HM). This is because the model could learn the regulation mechanisms of DNA methylation and transcription factors. Figure 5B illustrates the DNA methylation levels of *RNF212* and the attention weights of Dynamic LSTM. Surprisingly, the attention weight near TSS was high, and the region was unmethylated. The unmethylated promoter region enabled transcription factors to bind to the gene. Figure 5C shows all the possible binding transcription factors of the promoter region. These transcription factors were up-regulated especially in the HeLa cell compared to the other 17 cell lines (Figure 5D). Therefore, we can infer that *RNF212* could be highly expressed, despite weak signals of activation marks, thanks to the help of the “highly expressed” transcription factors, which bound to the unmethylated promoter region. As shown in the example, our multi-omics model reflected the multiple gene regulation mechanisms of histone marks, DNA methylation, and transcription factors.

### 3.4. Characterizing Cell-Type-Specific Gene Regulation Mechanisms

**Figure 6:**
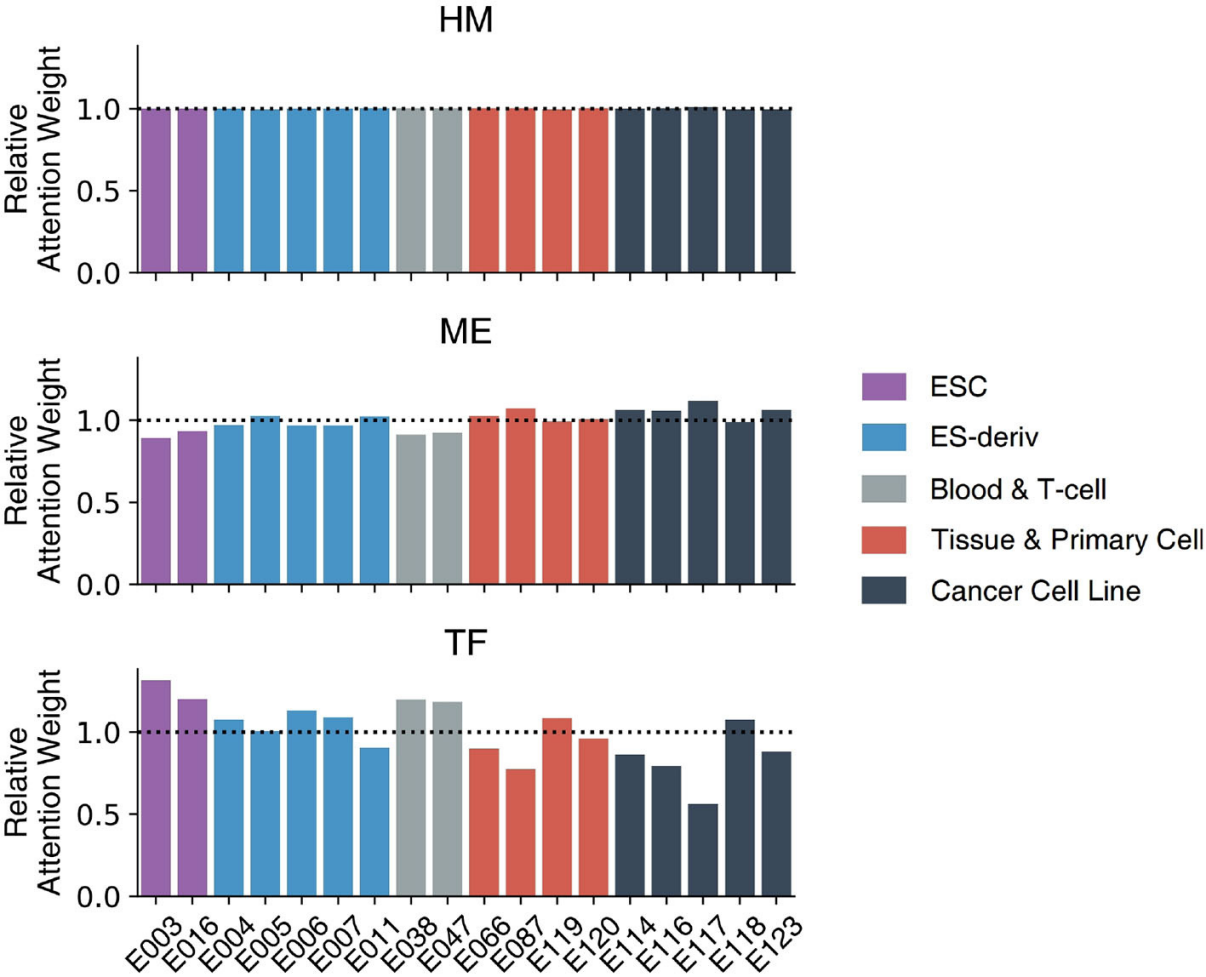
The weights of the Multi-Attention Block in 18 cell lines. Based on relative attention weights, the importance of histone marks was not significantly different among cell lines. Moreover, methylation and transcription factors showed quite different weights among each cell line. In case of methylation, cancer cell lines were more focused on methylation compared to other cell lines. In case of transcription factor, ESC type cell lines had higher attention weights than those of others.

Figure 6 demonstrates the weights of the Multi-Attention Block in 18 cell lines. Every weight is normalized by the average weight of 18 cell-lines in order to compare the importance of markers in each cell line. The attention weight of each feature shows how the model attends to the feature to predict gene expression level. The attention weight of each epigenetic marker therefore represents the importance of the markers in gene regulation. We compared the attention weights to reveal the important regulatory mechanisms in 18 cell lines and 5 cell types: ESC, ES-deriv, Blood & T-cell, Tissue & Primary Cell, and Cancer Cell Line.

There was no big difference in the weights of histone marks between the 18 cell lines. This is because histone modification plays a key role in the activation of genes, irrespective of cell type. Only after the chromatin structure of the gene is opened, can the gene be expressed. The higher AUC of the HM model (91.05) compare to that of the TF model (73.54) or the ME model (79.54), supports the importance of histone marks.

In contrast, weights of DNA methylation or transcription factors vary among cell types. In other words, DNA methylation and transcription factors determine the cell-type-specific gene regulatory mechanism. In general, cancer cell lines showed high attention weights of DNA methylation. The result is intuitive because DNA methylation is important in the development of cancer (Wajed et al., 2001; Kulis and Esteller, 2010). The abnormal patterns of methylation can inhibit gene expression and increase the probability of mutation (Wajed et al., 2001; Kulis and Esteller, 2010). It is commonly known that the hypermethylation of CpG islands inactivates tumor suppressor genes. Moreover, global hypomethylation significantly contributes to genome instability and aberrant gene expression.

In addition, embryonic stem cells showed the high attention weights of transcription factors. This reflects the crucial role of transcription factors in determining the fate of stem cells between self-renewal and differentiation. Transcriptional circuitry involving transcription factors like *OCT4, SOX2*, and *NANOG* is well-known to be a core regulatory mechanism of stem cells to maintain their stemness (Pan et al., 2002; Li, 2010). Furthermore, the significance of transcriptional regulation in embryonic stem cells has been highlighted since the prominent discovery, showing that ectopic overexpression of four essential transcription factors (*OCT4, SOX2, KLF4, MYC*), which are often referred to as “Yamanaka factors,” are sufficient to induce the pluripotency of somatic cells.

**Figure 7:**
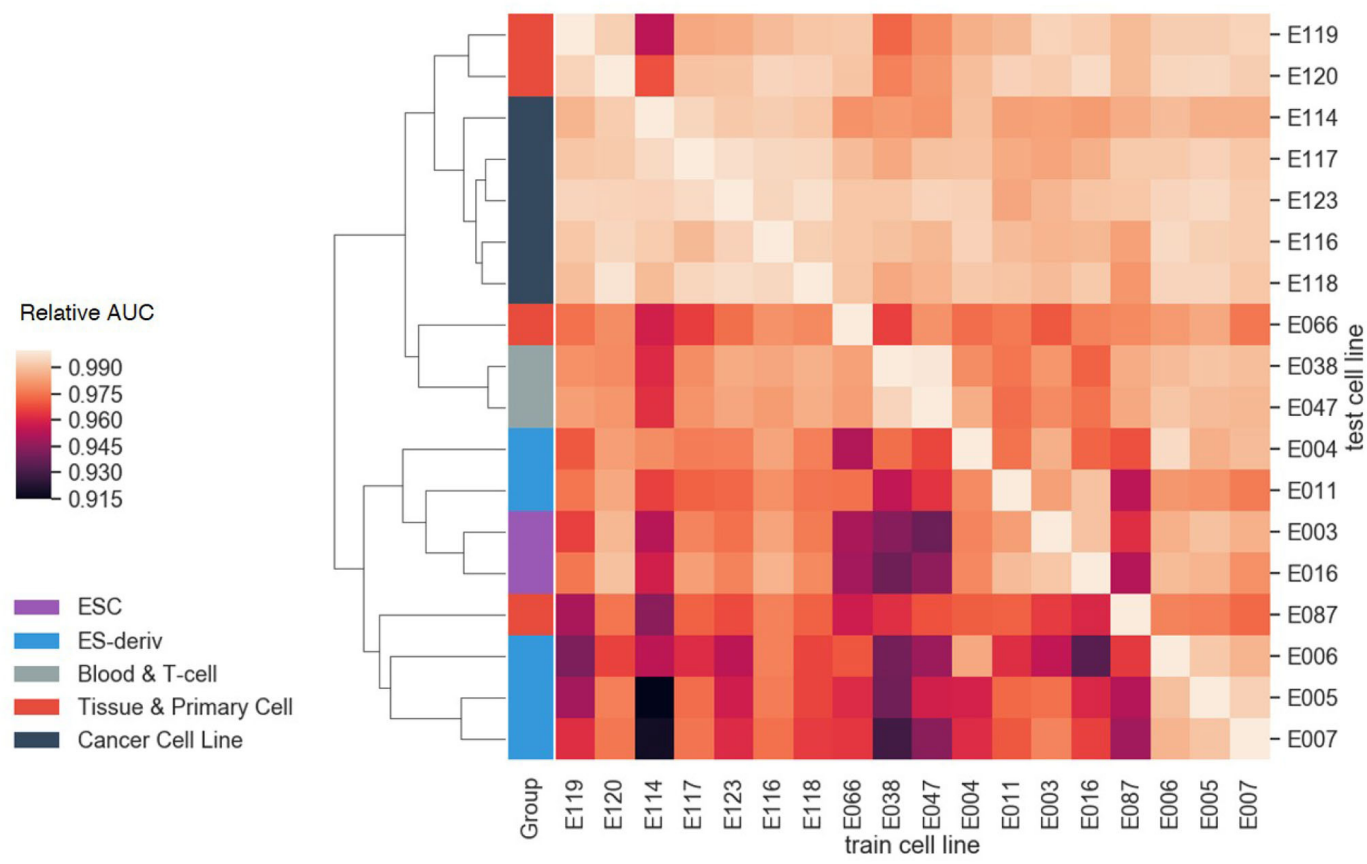
The compatibility test results between cell lines. The AUC values of each test cell line were normalized with the AUC value of the model trained on that cell line. As a result of hierarchical clustering, cell lines in the same group showed similar AUC patterns. This result highlights the transferability between the models in the same group.

Furthermore, we evaluated the cell-type-specificity and compatibility of our model, by training on one cell line and testing on other cell lines. For each cell line, the greatest AUC of the model was achieved when the model was trained on the cell line, demonstrating the cell-type-specificity. Moreover, it is notable that cell lines in the same group showed similar AUC patterns (Figure 7). By performing hierarchical clustering with the Euclidean distance, cell lines in the same group were clustered together. The result highlights the transferability between the models in the same group, i.e., *transfer learning*. In other words, each cell line can be explained well by the model of the other cell lines if they are in the same group. For instance, the blood and T-cell group, E038 and E047, showed the best AUC for each other's model. This is probably because the cell lines in the same group tend to have similar gene regulation mechanisms.

### 3.5. Identification of Enriched Genes: Case Studies on HeLa and K562

Performances of the multi-omics model on the HeLa cell line and K562 cell line were quite improved compared to AttentiveChrome (Supplementary Figure 5). In addition, the multi-omics model better captured cell line enriched genes that were obtained from the Human Protein Atlas (http://www.proteinatlas.org; Uhlen et al., 2017). In the case of the HeLa cell line, the multi-omics model predicted 12 genes correctly among 20 enriched genes, while 9-10 genes were predicted correctly by the HM, TF+HM, and ME+HM models (Supplementary Table 1). On the other hand, in the case of the K562 cell line, 38 out of 62 genes were predicted correctly with the multi-omics model. Similar to the HeLa cell case, other models showed poor performances (34-37 genes, Supplementary Table 2). The number of correctly predicted HEG by each model is summarized in Supplementary Figures 6, 7 for HeLa and K562, respectively.

**Figure 8:**
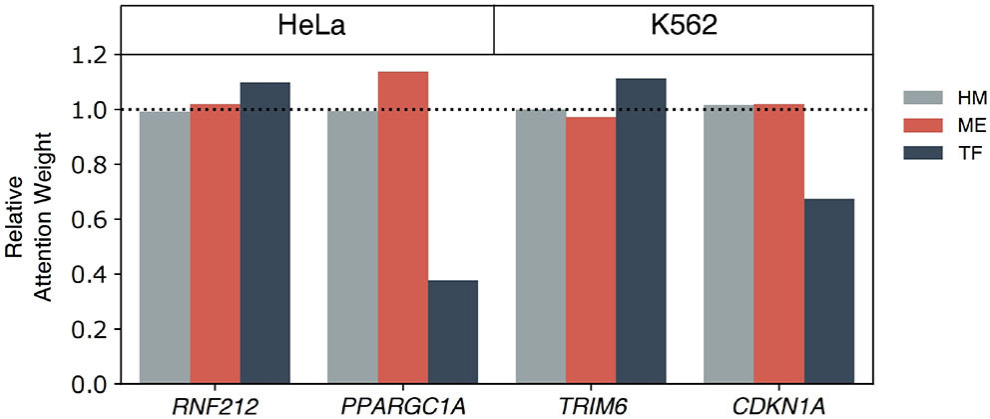
The epigenetic mechanisms of highly expressed genes in the HeLa and K562 cell lines. Among correctly predicted highly expressed genes by the TF+ME+HM model, four genes are selected for a further case study of epigenetic gene regulation mechanism: *RNF212* and *PPARGC1A* for HeLa, and *TRIM6* and *CDKN1A* for the K562 cell line. To elucidate the importance of each omics on the gene regulation, the relative attention weights of the Multi-Attention Block were used.

We further investigated functions and epigenetic regulation mechanisms of cell-type enriched genes on the HeLa and K562 cell lines (Figure 8). *RNF212* was one of the HeLa cell enriched genes and was predicted correctly with the multi-omics only model. *RNF212* creates a cellular memory of DNA damage by tagging the lingering breaks (Qiao et al., 2018) and is known as a prognostic marker in cervical cancer in The Human Protein Atlas http://www.proteinatlas.org. The TF+HM and ME+HM model failed to predict the expression level of *RNF212*, while the TF+ME+HM model predicted it as an expressed gene. This result therefore implies that the expression of *RNF212* may be modulated by DNA methylation and transcription factors. It is also shown in the weights of the Multi-Attention Block in Figure 8.

*PPARGC1A* was also predicted correctly by the multi-omics model of the HeLa cell line. *PPARGC1A* belongs to the PCG-1 family that is associated with the regulation of mitochondrial biogenesis, promoting cell growth, proliferation, and evasion of the apoptosis signal (Lin et al., 2005; Jones et al., 2012). In particular, *PPARGC1A* modulates telomere function and the DNA damage mechanism in diabetes and cardiovascular disease (Lai et al., 2008; Xiong et al., 2015). Interestingly, the TF+ME+HM and ME+HM model, but not the TF+HM model, correctly predicted expression of the gene. In Figure 8, methylation was relatively more highlighted than other omics data. According to this observation, we speculated that methylation is one of the main regulators of *PPARGC1A*. This epigenetic regulation was already reported in other tissues such as brown adipose tissue or skeletal muscle tissue (Gillberg et al., 2013, 2014; Gill and La Merrill, 2017).

In the case of the K562 cell line, the *TRIM6* gene was captured by the multi-omics model. It belongs to the Tripartite motif (TRIM) family which is related to the cancer stem cell self-renewal process. *TRIM6*, more specifically, directly interacts with the *MYC* gene to modulate stem cell differentiation (Jaworska et al., 2019). Attention weights of TF were relatively higher than the weights of ME. Besides the TF+ME+HM model, the TF+HM only model predicted the activation of genes correctly. Therefore, it is thought that HM and TF co-regulate the expression of *TRIM6*.

Lastly, *CDKN1A*, also known as p21, is a kind of tumor suppressor gene. *CDKN1A* plays a crucial role in regulating cell cycles to prevent cancer progression. In Figure 8, histone and methylation were relatively highlighted. Based on this observation, we thought that methylation might be a key factor in epigenetic regulation of *CDKN1A*. In addition to the TF+ME+HM model, the ME+HM model also predicted correctly. From previous studies, expression of *DNMT1* and *CDKN1A* showed a negative regulation mechanism on chronic myelogenous leukemia (Kaufman-Szymczyk et al., 2019). It was also reported that *DNMT3B* knock-down induced up-regulation of a number of tumor suppressor genes including *CDKN1A* (Poole et al., 2017).

Based on the case study of enriched genes of the HeLa and K562 cell line, we could investigate the epigenetic regulatory mechanisms of gene expressions by the weights of the Multi-Attention Blocks. Although it was possible to infer the involvement of histone marks, DNA methylation, and transcription factor for each gene, and to analyze their importance, there are other epigenetic and transcriptional factors that regulate gene expression. MicroRNA (miRNA) is one of the famous epigenetic factors that was not included in the model. MiRNAs are actually genes that are controlled by epigenetic mechanisms and TFs. For example, EWS is known to regulate Drosha, which controls biogenesis of miRNA (Kim et al., 2014). miRNA can then affect the transcription and translation of genes. To study the effects of miRNAs, we collected the genes that were correctly predicted by the TF+ME+HM model, not by the HM model in the HeLa cell line and 275 genes were selected as candidate genes. Using a biomedical literature search platform, BEST (Lee et al., 2016), 14 genes were related to miRNA in the context of the HeLa cell line, cervical cancer, or ovarian cancer (Supplementary Table 3). For example, the expression of LPAR2 was repressed by miR-377, and oncogenic processes such as cell proliferation or migration are known to be repressed by that inhibition mechanism (Zhang et al., 2020). As another example, ITGB1 was targeted by miR-183. It is known that miR-183 may play a role in tumor suppressors, such as the inhibition of cell invasion or the decrease of migration capacities of HeLa cells (Li et al., 2010). Incorporation of miRNA in our deep learning model can certainly be helpful in understanding complex gene regulation mechanisms. We plan to investigate how roles of miRNA can be seamlessly integrated into our deep learning model.

## 4. Conclusion

In summary, the proposed model learned cell-type-specific gene regulation mechanisms through Multi-Attention based deep learning strategies. To the best of our knowledge, the model is the first of its kind to use multiple epigenetic and transcriptional markers for predicting gene expressions. Our model achieved higher prediction accuracy than the state-of-the-art model. Additionally, the proposed method provided useful insight into cell-type-specific gene regulation mechanisms. Specifically, the weights of the Multi-Attention Block revealed the relative importance of each marker in the specific cell line. Lastly, we identified the mechanism of enriched genes in HeLa and K562 cell lines.

Our model investigated the roles of three markers: histone marks, DNA methylation, and transcription factors. However, the gene regulatory network may also involve additional epigenetic and transcriptional markers such as microRNA, competing endogenous RNA, or long non-coding RNA. Thus, future studies on other epigenetic markers need to be conducted.

## Data Availability Statement

Publicly available datasets were analyzed in this study. This data can be found here: https://egg2.wustl.edu/roadmap/data/byDataType/rna/expression/, https://egg2.wustl.edu/roadmap/data/byDataType/dnamethylation/, and https://egg2.wustl.edu/roadmap/data/byFileType/alignments/consolidated/.

## Author Contributions

SK conceived the experiment. MK and SL conducted the experiment and drafted the manuscript. MK, SL, and DL processed data and analyzed results. All authors read and approved the final manuscript.

## Conflict of Interest

The authors declare that the research was conducted in the absence of any commercial or financial relationships that could be construed as a potential conflict of interest.
