# Supplementary material for: Learning Cell-Type-Specific Gene Regulation Mechanisms by Multi-Attention Based Deep Learning With Regulatory Latent Space
Source: Front Genet. 2020 Sep 30;11:869. doi: 10.3389/fgene.2020.00869 (PMC7561362; doi:10.3389/fgene.2020.00869)
Supplement: Supplementary file 1 [file Data_Sheet_1.PDF]

Supplementary Material: Learning Cell-Type-Specific Gene Regulation Mechanisms by Multi-Attention Based Deep Learning with Regulatory Latent Space

1 SUPPLEMENTARY FIGURES AND TABLES

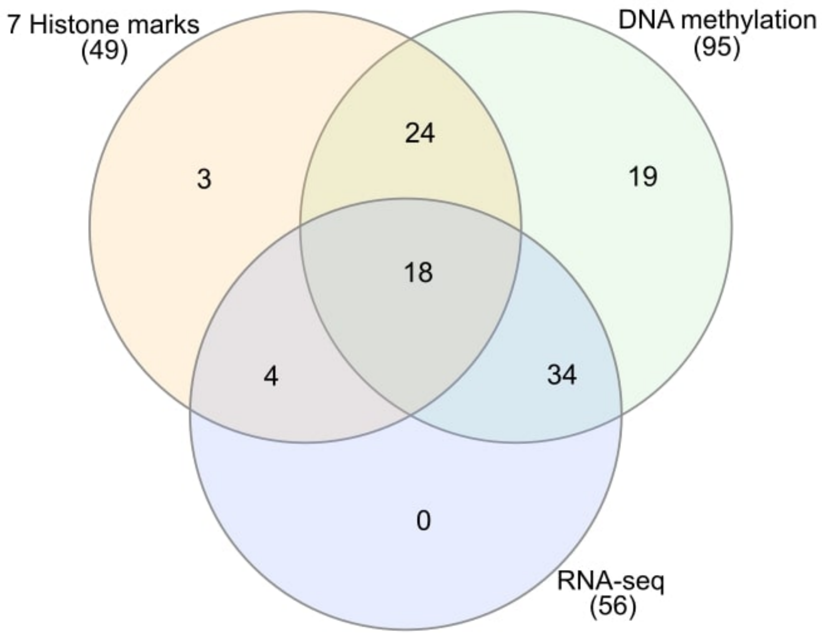

Figure S1: The number of available cell lines for 7 histone marks, DNA methylation, and RNA-seq.

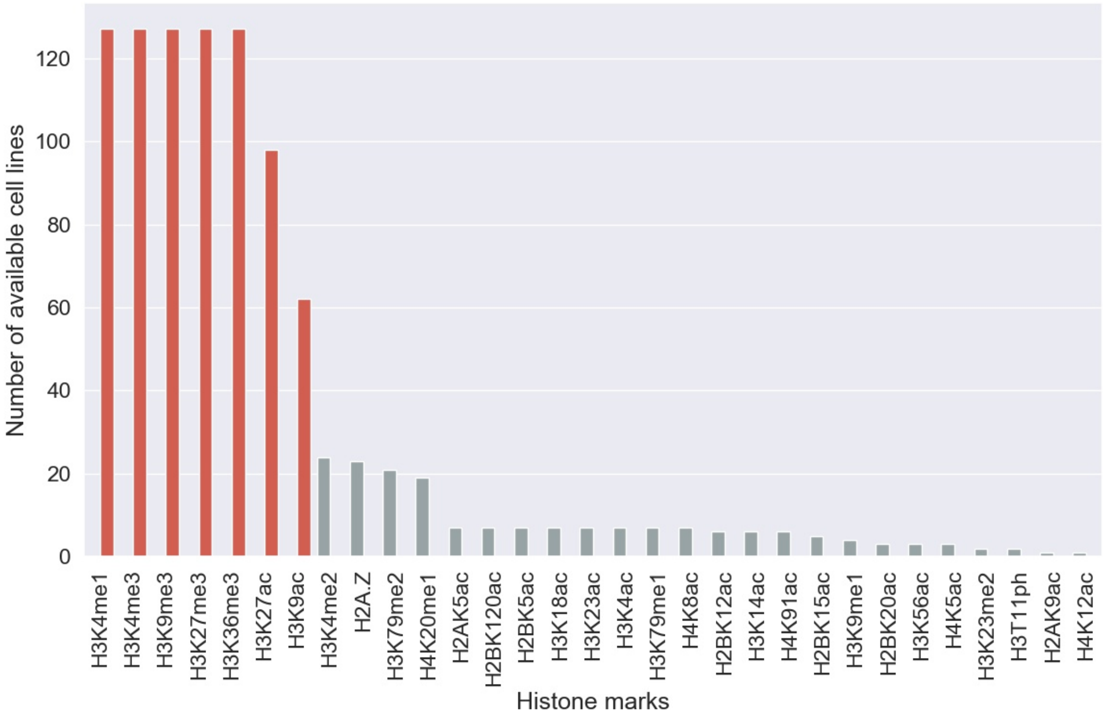

Figure S2: The number of available cell lines for each histone mark.

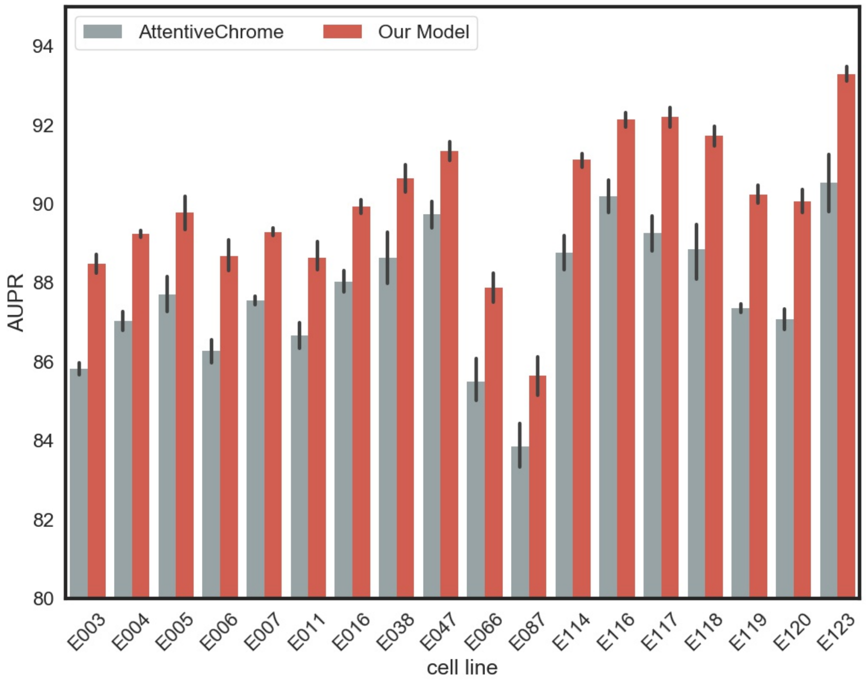

Figure S3: AUPR for our model and a baseline model, AttentiveChrome, for 18 cell lines.

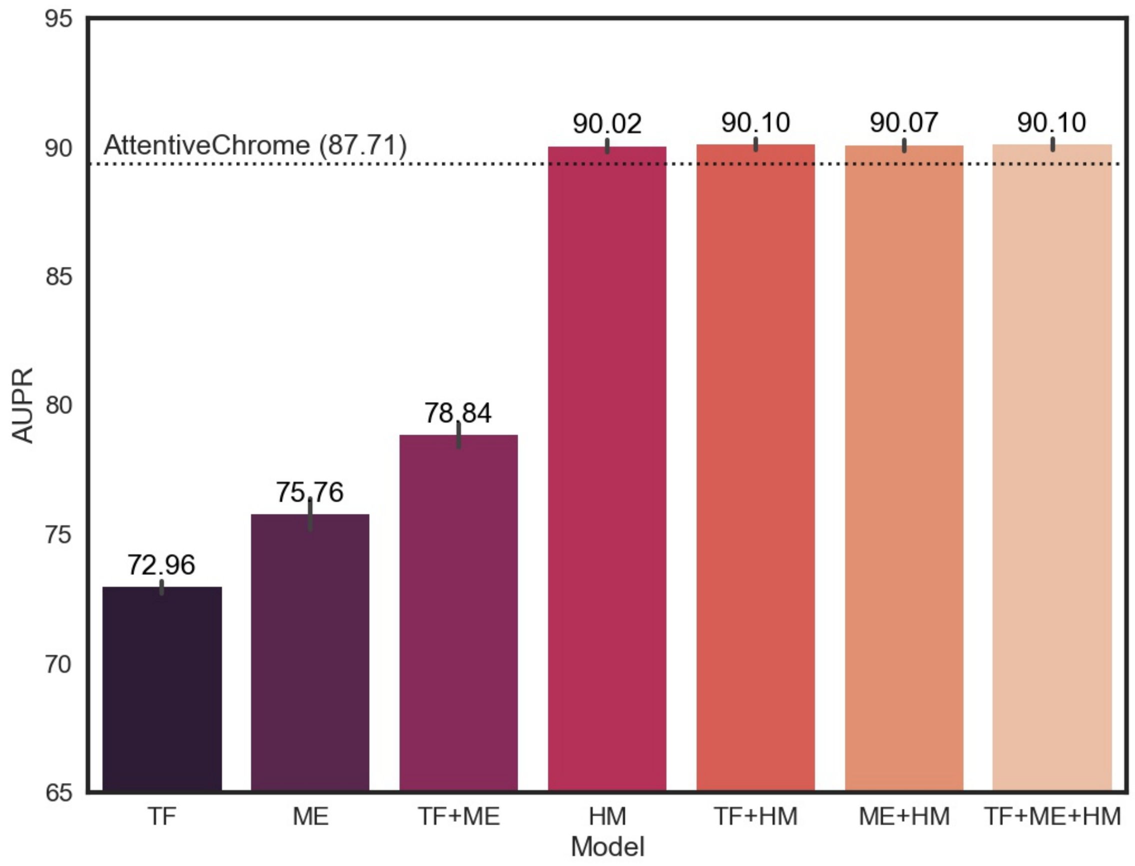

Figure S4: Average AUPR of 18 cell lines for different subsets of multi-omics features.

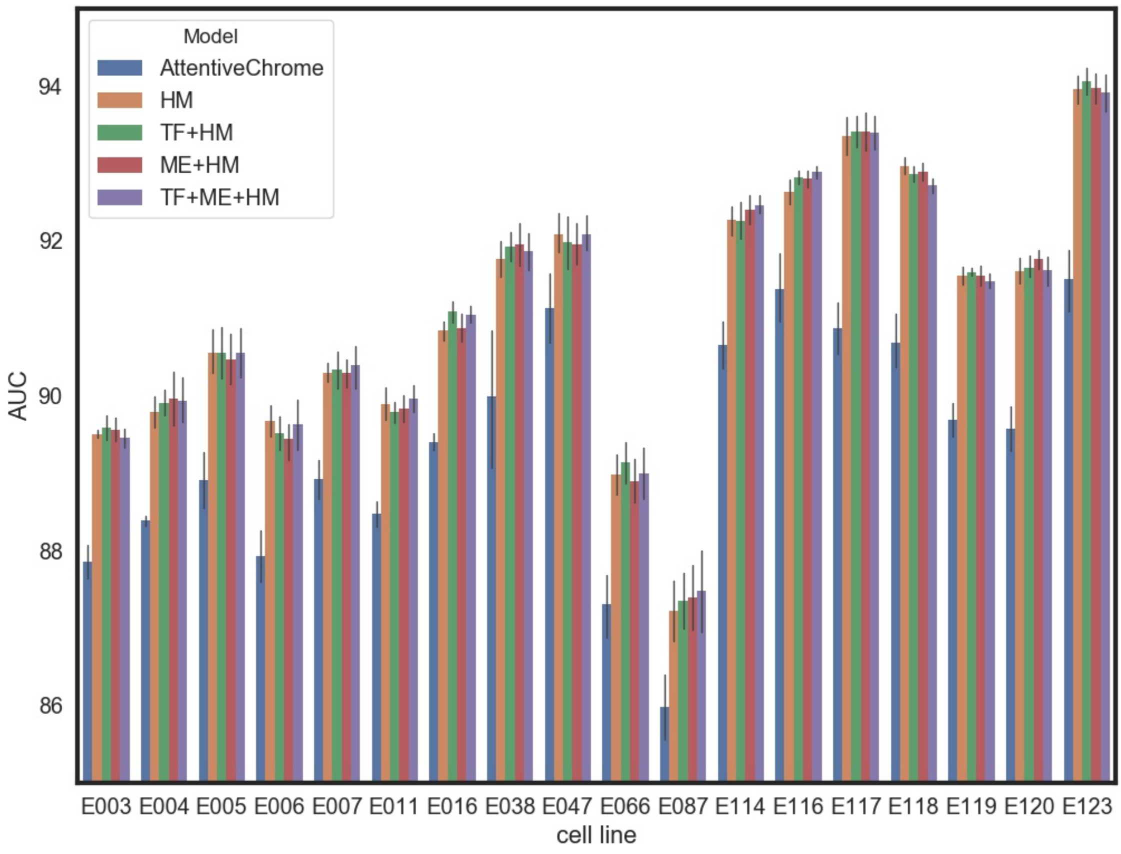

Figure S5: AUC of five models for all cell lines.

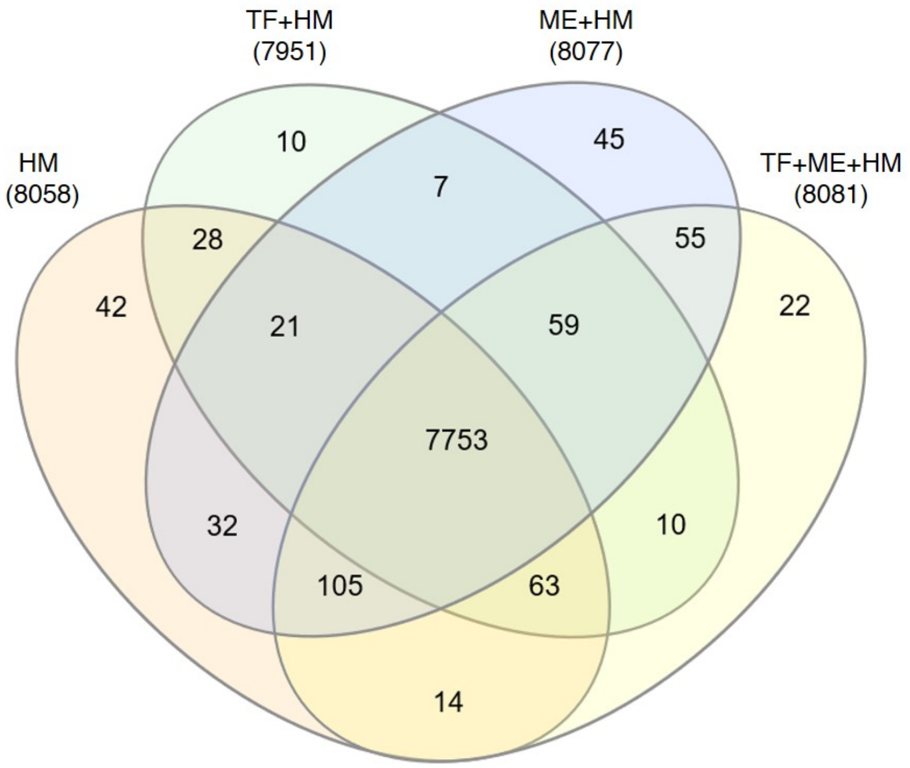

Figure S6: The number of correctly predicted HEG by each model in HeLa cell line.

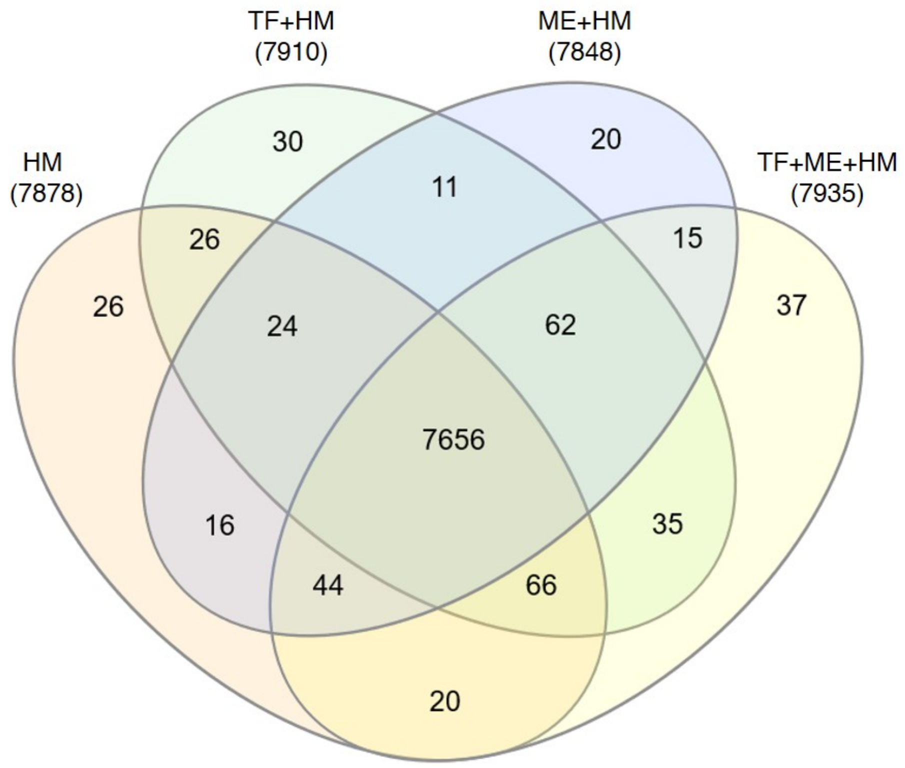

Figure S7: The number of correctly predicted HEG by each model in K562 cell line.

Table S1. The prediction of each model for enriched genes in HeLa cell line.

		Attentive Chrome	HM	ME+HM	TF+HM	TF+ME+HM
Enriched	GLP2R					
	SPINK13			✓	✓	✓
	COL25A1	✓	✓	✓	✓	✓
	SLF1	✓	✓	✓	✓	✓
	PDE2A					
	KLHL1					
Group Enriched	SLC12A3	✓	✓	✓	✓	✓
	KCNH5					
	C4BPB	✓	✓	✓	✓	✓
	RNF212					✓
	DHRS7C			✓		
	GPC5	✓	✓	✓	✓	✓
	USH1C		✓			✓
	IGSF10					
	C11orf86	✓	✓	✓	✓	✓
	ENO3	✓	✓	✓	✓	✓
	BEGAIN	✓				
	FOLR1					
	PDE8B					
	SAMD11	✓	✓	✓	✓	✓
Number of genes		9	9	10	9	12

Table S2. The prediction of each model for enriched genes in K562 cell line.

		Attentive Chrome	HM	ME+HM	TF+HM	TF+ME+HM
Enriched	STAR	✓	✓	✓	✓	✓
	SPTA1	✓	✓	✓	✓	✓
	NT5DC4					
	PRAME	✓	✓	✓	✓	✓
	MS4A4A					
	HBG1	✓	✓	✓	✓	✓
	C1orf100					
	CT55	✓	✓	✓	✓	✓
	PPIL2	✓	✓	✓	✓	✓
	TSPYL6	✓	✓	✓	✓	✓
	HBE1	✓	✓	✓	✓	✓
	HBZ		✓		✓	✓
	C12orf40	✓	✓	✓	✓	✓
	PKLR	✓	✓	✓	✓	✓
	HEMGN	✓				✓
Group Enriched	STEAP1B					
	GYPE	✓	✓	✓	✓	✓
	ANK1					
	OR51B4		✓	✓	✓	✓
	ALAS2	✓	✓	✓	✓	✓
	SEC14L6					
	HRC	✓	✓	✓	✓	✓
	GP6	✓	✓	✓	✓	✓
	SH3GL3	✓	✓	✓	✓	✓
	NOSTRIN					
	KCNH2	✓	✓	✓	✓	✓
	C17orf99	✓	✓	✓	✓	✓
	HTR1F					
	C2orf88					
	HBA1		✓	✓	✓	✓
	SLC25A21-AS1	✓	✓	✓	✓	✓
	HBG2					
	CR1L	✓	✓	✓	✓	✓
	HBQ1	✓	✓	✓	✓	✓
	RHD		✓	✓	✓	✓
	GAGE12J			✓	✓	
	CLIC2	✓	✓	✓	✓	✓
	HBA2		✓	✓		
	TFR2	✓	✓	✓	✓	✓
	ALDH1A2					
	FAM83A	✓				
	NBPF4					
	RHAG	✓	✓	✓	✓	✓
	TUBAL3					
	PPP1R14A	✓	✓	✓	✓	✓
	MS4A3					
	NAA11	✓	✓	✓	✓	✓
	GAGE12F					
	MAGEC2		✓	✓	✓	✓
	KEL	✓	✓	✓	✓	✓
	CTAG2				✓	
	MFSD2B					✓
	AMHR2	✓				
	APLNR					
	MRAP2	✓	✓	✓	✓	✓
	MAGEB1					
	ASIC4	✓	✓	✓	✓	✓
	CFAP161					
	SLC25A21	✓	✓	✓	✓	✓
	MEIOB					
	UBXN10	✓	✓	✓	✓	✓
	SAMSN1	✓	✓	✓	✓	✓
Number of genes		34	37	36	37	38

Table S3. Literature survey on miRNAs related to correctly predicted genes by only TF+ME+HM model

Gene	Related biological terms		
	HeLa	Cervival	Ovarian
LPAR2	miR-377 (Zhang et al., 2020)	miR-377 (Zhang et al., 2020)	
ITGB1	miR-183 (Li et al., 2010)	miR-183 (Zhang et al., 2018) miR-361 (Yang and Xie, 2020)	
APOBEC3B		miR-34b (Revathidevi et al., 2016)	
COL4A1		miR-29a (Zhao et al., 2019b)	
SLIT2		miR-let-7c, miR-34a miR-281-1, miR-23b, miR-145, miR-146a (Barzon et al., 2014)	
TP53INP1		miR-155 (Li et al., 2019) miR-15a (Zhao et al., 2019a) miR-214 (Sen et al., 2020) miR-17 (Wei et al., 2012)	miR-569 (Chaluvally-Raghavan et al., 2014)
TIMP3		miR-21 (Zhang et al., 2018) miR-346 (Sun et al., 2019) miR-221 (Fu et al., 2018)	miR-191 (Dong et al., 2015) miR-98 (Panda et al., 2012)
LPP			miR-26b (Tang and Luo, 2018)
BMF			miR-221 (Xie et al., 2018)
UTP14C			miR-145 (Rohozinski et al., 2012)
SPINT2			miR-let-7b, miR-29a miR-30d, miR-205, miR-23b (Yamamoto et al., 2018)
SIRT7			miR-17 (Ding et al., 2020)
GRAMD1B			miR-193b (Ziliak et al., 2012)
CORIN			miR-302b, miR-204, miR-211 (Zou et al., 2020)

REFERENCES

Barzon, L., Cappellesso, R., Peta, E., Militello, V., Sinigaglia, A., Fassan, M., et al. (2014). Profiling of expression of human papillomavirus–related cancer mirnas in penile squamous cell carcinomas. *The American journal of pathology* 184, 3376–3383

Chaluvally-Raghavan, P., Zhang, F., Pradeep, S., Hamilton, M. P., Zhao, X., Rupaimoole, R., et al. (2014). Copy number gain of hsa-mir-569 at 3q26. 2 leads to loss of tp53inp1 and aggressiveness of epithelial cancers. *Cancer Cell* 26, 863–879

Ding, C., Zhu, L., Shen, H., Lu, J., Zou, Q., Huang, C., et al. (2020). Exosomal mirna-17-5p derived from human umbilical cord mesenchymal stem cells improves ovarian function in premature ovarian insufficiency by regulating sirt7. *STEM CELLS*

Dong, M., Yang, P., and Hua, F. (2015). Mir-191 modulates malignant transformation of endometriosis through regulating timp3. *Medical science monitor: international medical journal of experimental and clinical research* 21, 915

Fu, F., Wang, T., Wu, Z., Feng, Y., Wang, W., Zhou, S., et al. (2018). Hmga1 exacerbates tumor growth through regulating the cell cycle and accelerates migration/invasion via targeting mir-221/222 in cervical cancer. *Cell death & disease* 9, 1–17

Li, G., Luna, C., Qiu, J., Epstein, D. L., and Gonzalez, P. (2010). Targeting of integrin β1 and kinesin 2α by microrna 183. *Journal of Biological Chemistry* 285, 5461–5471

Li, N., Cui, T., Guo, W., Wang, D., and Mao, L. (2019). Mir-155-5p accelerates the metastasis of cervical cancer cell via targeting tp53inp1. *OncoTargets and therapy* 12, 3181

Panda, H., Chuang, T.-D., Luo, X., and Chegini, N. (2012). Endometrial mir-181a and mir-98 expression is altered during transition from normal into cancerous state and target pgr, pgrmc1, cyp19a1, ddx3x, and timp3. *The Journal of Clinical Endocrinology & Metabolism* 97, E1316–E1326

Revathidevi, S., Manikandan, M., Rao, A. K. D. M., Vinothkumar, V., Arunkumar, G., Rajkumar, K. S., et al. (2016). Analysis of apobec3a/3b germline deletion polymorphism in breast, cervical and oral cancers from south india and its impact on mirna regulation. *Tumor Biology* 37, 11983–11990

Rohozinski, J., Edwards, C. L., and Anderson, M. L. (2012). Does expression of the retrogene utp14c in the ovary pre-dispose women to ovarian cancer? *Medical hypotheses* 78, 446–449

Sen, P., Ghosal, S., Hazra, R., Arega, S., Mohanty, R., Kulkarni, K. K., et al. (2020). Transcriptomic analyses of gene expression by crispr knockout of mir-214 in cervical cancer cells. *Genomics* 112, 1490–1499

Sun, Q., Yang, Z., Li, P., Wang, X., Sun, L., Wang, S., et al. (2019). A novel mirna identified in grsf1 complex drives the metastasis via the pik3r3/akt/nf-κb and timp3/mmp9 pathways in cervical cancer cells. *Cell death & disease* 10, 1–16

Tang, A. and Luo, J. (2018). Effects of 7-difluoromethy-5, 4'-dimethoxygenistein on stress urinary incontinence model in rats and its mechanisms. *Zhong nan da xue xue bao. Yi xue ban= Journal of Central South University. Medical sciences* 43, 260–267

Wei, Q., Li, Y.-X., Liu, M., Li, X., and Tang, H. (2012). Mir-17-5p targets tp53inp1 and regulates cell proliferation and apoptosis of cervical cancer cells. *IUBMB life* 64, 697–704

Xie, X., Huang, Y., Chen, L., and Wang, J. (2018). mir-221 regulates proliferation and apoptosis of ovarian cancer cells by targeting bmf. *Oncology letters* 16, 6697–6704

Yamamoto, C. M., Oakes, M. L., Murakami, T., Muto, M. G., Berkowitz, R. S., and Ng, S.-W. (2018). Comparison of benign peritoneal fluid-and ovarian cancer ascites-derived extracellular vesicle rna biomarkers. *Journal of ovarian research* 11, 20

Yang, W. and Xie, T. (2020). Hsa_circ_cspp1/mir-361-5p/itgb1 regulates proliferation and migration of cervical cancer (cc) by modulating the pi3k-akt signaling pathway. *Reproductive Sciences* 27, 132–144

Zhang, W., Zhang, M., Liu, L., Jin, D., Wang, P., and Hu, J. (2018). Microrna-183-5p inhibits aggressiveness of cervical cancer cells by targeting integrin subunit beta 1 (itgb1). *Medical Science Monitor: International Medical Journal of Experimental and Clinical Research* 24, 7137

Zhang, Y., Liu, Y., Guo, X., Hu, Z., and Shi, H. (2020). Interfering human papillomavirus e6/e7 oncogenes in cervical cancer cells inhibits the angiogenesis of vascular endothelial cells via increasing mir-377 in cervical cancer cell-derived microvesicles. *OncoTargets and therapy* 13, 4145

Zhao, X., Tang, H., Yang, J., Gu, X., Wang, S., and Ding, Y. (2019a). Microrna-15a-5p down-regulation inhibits cervical cancer by targeting tp53inp1 in vitro. *European review for medical and pharmacological sciences* 23, 8219–8229

Zhao, Y.-Y., Duan, R.-N., Ji, L., Liu, Q.-J., and Yan, C.-Z. (2019b). Cervical spinal involvement in a chinese pedigree with pontine autosomal dominant microangiopathy and leukoencephalopathy caused by a 3 untranslated region mutation of col4a1 gene. *Stroke* 50, 2307–2313

Ziliak, D., Gamazon, E. R., LaCroix, B., Im, H. K., Wen, Y., and Huang, R. S. (2012). Genetic variation that predicts platinum sensitivity reveals the role of mir-193b* in chemotherapeutic susceptibility. *Molecular cancer therapeutics* 11, 2054–2061

Zou, X., Wang, J., Qu, H., Lv, X., Shu, D., Wang, Y., et al. (2020). Comprehensive analysis of mirnas, lncrnas, and mrnas reveals potential players of sexually dimorphic and left-right asymmetry in chicken gonad during gonadal differentiation. *Poultry Science*
